# TSA Promotes CRISPR/Cas9 Editing Efficiency and Expression of Cell Division-Related Genes from Plant Protoplasts

**DOI:** 10.3390/ijms22157817

**Published:** 2021-07-22

**Authors:** Seung Hee Choi, Myoung Hui Lee, Da Mon Jin, Su Ji Ju, Woo Seok Ahn, Eun Yee Jie, Ji Min Lee, Jiyoung Lee, Cha Young Kim, Suk Weon Kim

**Affiliations:** 1 Biological Resource Center, Korea Research Institute of Bioscience and Biotechnology, 181 Ipsingil, Jeongeup-si 56212, Korea; csh@kribb.re.kr (S.H.C.); ya6930@naver.com (D.M.J.); suji@kribb.re.kr (S.J.J.); dntjr0412@kribb.re.kr (W.S.A.); jeannie@kribb.re.kr (E.Y.J.); jimin1215@kribb.re.kr (J.M.L.); jiyoung1@kribb.re.kr (J.L.); kimcy@kribb.re.kr (C.Y.K.); 2 National Institute of Crop Science, RDA, Wanju 55365, Korea; mhlee17@korea.kr; 3 Department of Bioenergy Science and Technology, Chonnam National University, Gwangju 61186, Korea; 4 Department of Applied Plant Science, Chonnam National University, Gwangju 61186, Korea

**Keywords:** trichostatin A, genome editing efficiency, histone acetylation, chromatin de-condensation, plant protoplasts, lettuce, tobacco

## Abstract

Trichostatin A (TSA) is a representative histone deacetylase (HDAC) inhibitor that modulates epigenetic gene expression by regulation of chromatin remodeling in cells. To investigate whether the regulation of chromatin de-condensation by TSA can affect the increase in the efficiency of Cas9 protein-gRNA ribonucleoprotein (RNP) indel formation from plant cells, genome editing efficiency using lettuce and tobacco protoplasts was examined after several concentrations of TSA treatments (0, 0.1, 1 and 10 μM). RNP delivery from protoplasts was conducted by conventional polyethylene glycol (PEG) transfection protocols. Interestingly, the indel frequency of the *SOC1* gene from TSA treatments was about 3.3 to 3.8 times higher than DMSO treatment in lettuce protoplasts. The TSA-mediated increase of indel frequency of the *SOC1* gene in lettuce protoplasts occurred in a concentration-dependent manner, although there was not much difference. Similar to lettuce, TSA also increased the indel frequency by 1.5 to 1.8 times in a concentration-dependent manner during *PDS* genome editing using tobacco protoplasts. The MNase test clearly showed that chromatin accessibility with TSA treatments was higher than that of DMSO treatment. Additionally, TSA treatment significantly increased the level of histone H3 and H4 acetylation from lettuce protoplasts. The qRT-PCR analysis showed that expression of cell division-related genes (*LsCYCD1-1*, *LsCYCD3-2*, *LsCYCD6-1*, and *LsCYCU4-1*) was increased by TSA treatment. These findings could contribute to increasing the efficiency of CRISPR/Cas9-mediated genome editing. Furthermore, this could be applied for the development of useful genome-edited crops using the CRISPR/Cas9 system with plant protoplasts.

## 1. Introduction

The CRISPR/Cas system is an efficient genome editing technology following meganucleases, zinc-finger nucleases (ZFNs), and transcription activator-like effector nucleases (TALENs) [1,2,3]. The CRISPR/Cas9 system, consisting of the Cas9 protein derived from *Streptococcus pyogenes* and gRNA, is the most widely used system based on RNA-guided interference with DNA [4]. The gRNA is a small RNA that contains 20 nucleotides complementary to target sequences and an artificial fusion of a crRNA and a fixed transactivating crRNA for recruiting the Cas9 protein to direct the cleavage of DNA sequences adjacent to 5′-NGG-3′ protospacer-adjacent motifs (PAMs) [5]. As a result, the Cas9 protein cleaves the target region in a sequence-dependent manner, and a double-strand break (DSB) is generated and repaired in that region, resulting in genome modification.

The CRISPR/Cas system is actively used for genome editing in a variety of species, including plants [6,7,8]. In particular, the Cas9 protein-gRNA ribonucleoproteins (RNPs) system has received attention for its ability to reduce the possibility of the insertion of recombinant DNA into the host genome [9]. Advances in CRISPR/Cas genome editing have accelerated the improvement of crop traits and have produced transgene-free genome-edited plants in a short time [7]. The application of this system has been reported in various plants such as Arabidopsis, tobacco, rice, and wheat [1,2,10]. Traits mainly related to productivity, biological and abiotic stress resistance, and nutritional quality improvement are being manipulated by genome editing. Therefore, the technology for developing genome editing crops using CRISPR/Cas will help to develop active alternatives in the fields of plant-related global issues, such as strengthening the global food supply, responding to global warming, and sustainable agriculture.

To promote the efficiency of genome-edited plant development, there are three major ways to increase the efficiency of genome editing: efficient gRNA design, effective delivery systems, and increased gRNA accessibility to the target region through chromatin structural modification. In recent years, active research has been conducted on guidelines and software tools for effective CRISPR gRNA design [11,12,13]. The computational approaches have been developed for scoring guide RNAs to escape low cleavage efficiency and off-target effects [10]. Furthermore, the delivery of editing reagents to plant cells is a very critical step for the generation of genome-edited plants. Protoplast transfection, *Agrobacterium*-mediated transfer DNA (T-DNA) transformation, or particle bombardment are methods by which CRISPR-mediated editing reagents, including DNA, RNA, and RNPs can be delivered to plant cells. Direct transfection into the protoplast is used for the regeneration of transgene-free genome-edited plants, whereas *Agrobacterium*-mediated transformation and particle bombardment are normally used for the two major vector delivery methods for the production of edited plants [14]. Advances in efficient delivery systems will accelerate plant genome editing in the future. Lastly, increasing the efficiency of genome editing can be achieved by increased gRNA accessibility to the target region through chromatin structure. It was reported that the treatments of chromatin-modulating compounds induce histone hyperacetylation at the target sites, resulting in a significant increase in the efficiency of indel formation in a dose-response manner in mammalian cells [15]. In particular, it is known that TSA is effective in animal cells, but its effect has not been proven in plant cells.

Trichostatin A (TSA) is a representative histone deacetylase (HDAC) inhibitor that can bind to HDAC by inserting its long aliphatic chain into the catalytic active pocket, resulting in inhibition of the enzymatic activity of HDAC [16]. It was reported that TSA facilitates totipotency in the male gametophyte in *Brassica napus* [17], and HDAC inhibitors TSA and suberoylanilide hydroxamic acid (SAHA) improved the rate of microspore embryogenesis and the frequency of direct plant regeneration in pakchoi (*Brassica rapa* ssp. *chinensis* L.) [18]. Wheat microspore-derived embryogenesis and green plant regeneration were similarly activated by TSA treatment, suggesting that TSA leads to an increase in histone acetylation and global alteration of gene expression [19]. However, TSA’s effects on the genome editing efficiency of CRISPR/Cas9 have been not examined in plant protoplasts.

Therefore, the potential effects of TSA in CRISPR/Cas9 editing efficiency from lettuce and tobacco protoplasts was investigated by Deep-seq, MNase assay, Western blot analysis, and gene expression analysis by qRT-PCR in this study. Furthermore, to check for the presence of any inhibitory effect of TSA on callus formation from tobacco protoplasts, callus proliferation was observed. The experimental results suggest that TSA, a representative HDAC inhibitor, increases CRISPR/Cas9-mediated genome editing efficiency and expression of cell division-related genes in plants.

## 2. Results

### 2.1. Effects of TSA on Genome Editing Efficiency Using Cas9 Transfection from Protoplasts

**Figure 1:**
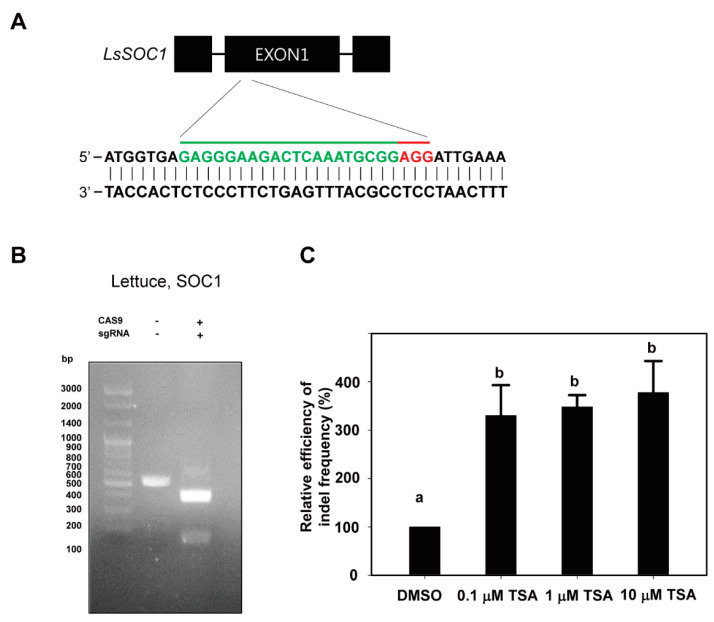
Changes in indel frequency according to TSA treatment in lettuce protoplasts. (**A**) Schematic illustration of the *LsSOC1* CRISPR/Cas9 target site. The target region is shown in green letters followed by PAM (NGG; red). (**B**) In vitro DNA cleavage assay using *LsSOC1* gRNA and Cas9 protein. The gRNA-mediated cleavage of target DNA in vitro is shown in agarose gel. The 546bp PCR fragment of the *LsSOC1* gene is used as a substrate for gRNA-Cas9 digestion. *LsSOC1* gRNA leads to a specific digestion of the target DNA, producing DNA fragments of 386 bp and 160 bp. (**C**) Relative efficiency of indel frequency (%) of at target site in protoplasts examined at 48 h after transfection of Cas9 and gRNAs as RNP complexes with DMSO or TSA treatment. The indel frequency of the DMSO treatment group was set to 100%, and those of the TSA treatment groups are shown relatively. Bars represent means ± SE (*n* = 3) of independent experiments. Different letters on the bars indicate significant differences between each treatment (ANOVA with the Duncan’s test, *p* < 0.05).

We tested whether a compound for epigenetic regulation, TSA, plays a stimulatory role in CRISPR/Cas9 genome editing during PEG transfection, especially by leading to chromatin structural modification. To prove this hypothesis, first of all, we selected the lettuce *SOC1* gene and tobacco *PDS* gene as targets for genome editing from protoplasts. We designed the gRNA of the lettuce *SOC1* gene using CRISPR RGEN Tools (http://www.rgenome.net/cas-designer/) (Figure 1A) and the gRNA of the tobacco *PDS* gene by referring to the previous study for gRNA design (Figure S1A). To investigate whether gRNA works properly, we examined whether it was cleaved under in vitro conditions. It was confirmed that Cas9–RNP complexes from lettuce were cleaved well at their target sites in the in vitro cleavage assay (Figure 1B).

To examine the effects of TSA, an inhibitor of HDACs, on genome editing efficiency of plant protoplasts with the Cas9 protein-gRNA RNP system, TSA was treated with different concentrations (0, 0.1, 1 and 10 μM) of freshly isolated protoplasts of lettuce immediately after PEG transfection. In the case of tobacco protoplasts, TSA was treated in the same manner as lettuce without 10 μM TSA treatment. TSA treatment significantly enhanced the indel frequency from lettuce protoplasts compared to DMSO treatment (Figure 1C and Table 1). In comparison with DMSO, TSA increased the indel frequency by 330%, 348% and 378% at concentrations of 0.1, 1 and 10 µM, respectively (Figure 1C). The indel frequency showed more than a three-times increase in all TSA treatments regardless of concentrations. The indel frequencies at the target site of *LsSOC1* gRNA for DMSO, 0.1, 1 and 10 μM TSA treatment were in the range of 0.9–2.7%, 4.0–8.6%, 3.4–9.9% and 4.5–9.6%, respectively. Although the indel frequencies differed in each experiment, the relative efficiencies of indel frequency showed a similar pattern in all three repeated experiments (Table 1). These results clearly represented that TSA has a stimulatory role in CRISPR/Cas9 editing. However, this was observed at a very low indel frequency in the only Cas9 protein without *LsSOC1* gRNA treatment.

Similar to lettuce protoplasts, the stimulatory effect of TSA on the increase in the indel frequency also showed a similar pattern to that of tobacco protoplasts (Figure S1B and Table S1). Treatment with 0.1 μM TSA and 1uM TSA increased the indel frequency by 149% and 184%, respectively, compared to DMSO control treatment. Although the increase in indel frequency was lower than that of lettuce, it was found that the indel frequency increased with 1 μM TSA treatment with statistical significance. A full list of the detected mutations is provided in the Supplementary excel data. After combining the results from the lettuce and tobacco protoplasts, we suggest that TSA treatment increases genome editing efficiency using plant protoplasts with the Cas9-RNP system.

### 2.2. Effects of TSA on the Mutation Patterns in Protoplast Cultures

**Figure 2:**
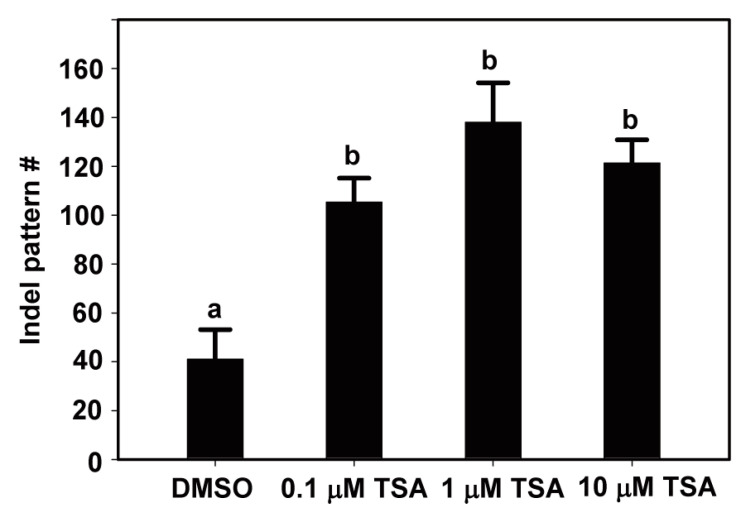
The number of indel patterns at the *LsSOC1* target loci in protoplasts examined at 48 h after transfection of Cas9 protein and gRNAs as RNP complexes with DMSO or TSA treatment. The total number of various indel patterns according to TSA treatment was analyzed by targeted deep sequencing.

**Figure 3:**
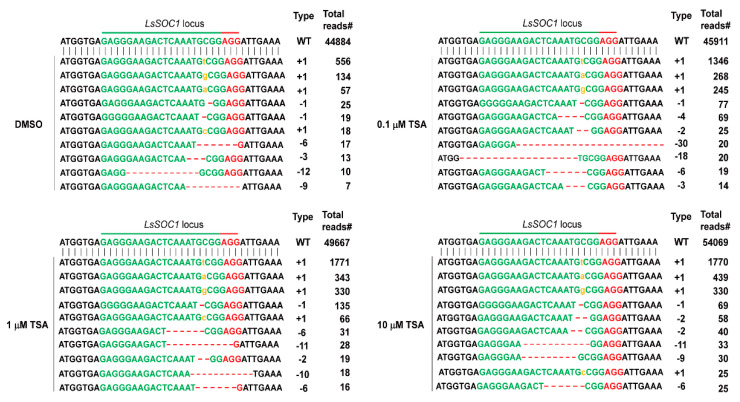
The representative indel types at *LsSOC1* target loci generated by delivery of Cas9 and gRNAs with DMSO or various concentrations of TSA treatment. An insertion of one base pair was the most common mutation pattern among the indel patterns induced by DMSO or various concentrations of TSA treatment at the *LsSOC1* loci. The target region is shown in green letters followed by PAM (NGG; red). Yellow lower-case letters mean inserted base, and red dash means nucleotide deletion.

Next, we analyzed the indel patterns induced by Cas9–RNP complexes with TSA treatment. Interestingly, TSA has not only increased the indel frequency but also induced more various indel mutation patterns from lettuce protoplasts (Figure 2). In the case of DMSO treatment, the indel mutation patterns at the target site of the *LsSOC1* gRNA were 41, but those of 0.1, 1 and 1 μM TSA were 105, 138 and 121, respectively (Figure 2). These results clearly show that TSA has a stimulatory role in the increase in indel mutation patterns from lettuce protoplasts using *LsSOC1* gRNA. The results suggest that TSA treatment induces more cleavage of the target by the Cas9 protein-gRNA RNPs, resulting in more mutations. An insertion of one base pair was the most common editing pattern among mutation patterns at the *LsSOC1* target loci in protoplasts treated with DMSO or TSA but showing a difference in mutation number (Figure 3). For example, in the case of 1 bp insertion, T insertion of target site, 0.1 μM, 1 μM and 10 μM TSA treatment yielded 1346, 1776 and 1770 reads, respectively, while DMSO treatment yielded 556 reads. Furthermore, different types of 1 bp insertions, A and G insertion, and short deletion are mainly found at the *LsSOC1* target loci in protoplasts treated with DMSO or TSA. The results are consistent with a previous study in which mutations caused by Cas9 during DSB repair were predominantly short deletions and 1 bp insertions [10,20]. The data suggest that although TSA treatment can lead to a variety of patterns, the main patterns are similar and lead to differences in the indel frequency.

### 2.3. Effects of TSA on the Structural Changes of Chromatin in Protoplast Cultures

**Figure 4:**
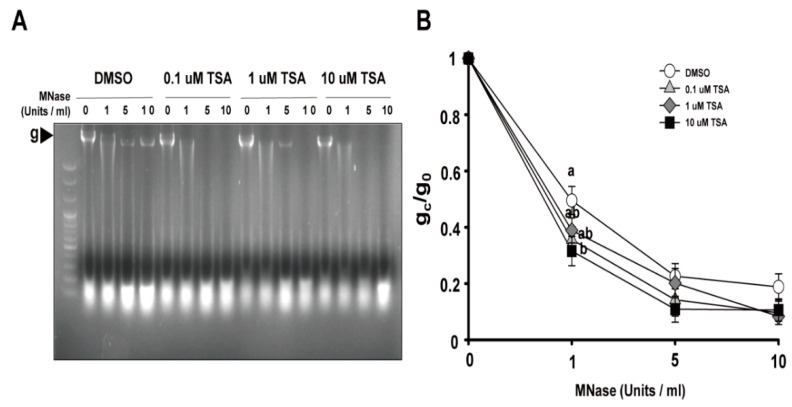
The effect of TSA on chromatin structures in lettuce protoplasts. (**A**) Representative image of chromatin digestion with micrococcal nuclease (MNase). The genomic band intensities with different concentrations of TSA were examined. The arrow means the genomic band without and with treatment with different concentrations of MNase. (**B**) Proportion of MNase-digested chromatin DNA resolved on agarose gels. The genomic band intensities without (g_0_) and with (g_c_) treatment with different concentrations of MNase were quantified, and the ratio of g_c_/g_0_ was used to represent the degree of chromatin relaxation. Bars represent means ± SE (*n* = 5) of independent experiments. Different letters on the bars indicate significant differences between each treatment (ANOVA with Duncan’s test, *p* < 0.05).

Since the packaging of eukaryotic DNA into chromatin restricts the ability of the Cas9 protein and gRNA to access their target and the recruitment of DNA damage repair machinery to DNA DSB sites cleaved by Cas9, the potential relationships between chromatin remodeling and genome editing have been explored in many organisms, from yeast to humans [21,22,23,24]. Accordingly, we investigated the effects of TSA on chromatin structure by a chromatin accessibility test using micrococcal nuclease (MNase) that cuts internucleosomal DNA. The ratios of intact genomic band intensities treated with different concentrations of MNase (g_c_) to the non-treated control (g_0_) represent the degree of chromatin relaxation. It can be seen that the higher the concentration of MNase in the DMSO-treated group, the lower the ratio of g_c_/g_0_ (Figure 4). Chromatin accessibility of TSA treatment groups was higher than that of DMSO treatment group, as revealed by the lower g_0_/g_c_ ratio. When treated with 1 units/mL MNase, the ratio decreased significantly according to the TSA concentration, which means that chromatin accessibility was higher. Although the difference between the TSA and DMSO treatment groups was small when 5 and 10 Units/mL MNase were treated, the ratio of g_c_/g_0_ of the TSA-treated group was lower than that of the DMSO-treated group, which means that chromatin accessibility was higher. These results indicate that TSA has a positive effect on the chromatin structural modification in protoplasts and may influence the ability of gRNA to access the target or the recruitment of DNA damage repair machinery to DNA DSB cleaved by Cas9 after PEG transfection.

### 2.4. Effects of TSA on Histone Acetylation in Protoplast Cultures

**Figure 5:**
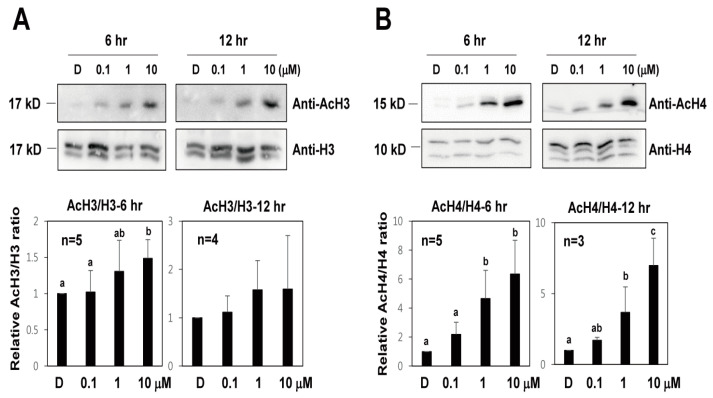
The effect of TSA on histone H3 and H4 acetylation level in lettuce protoplasts. Total protein extracts were obtained from lettuce protoplasts after 6 or 12 h after TSA treatments. Error bars represent SD (*n* = 3 or more). (**A**) H3 Histone acetylation levels were determined with a Western blot using an anti-H3 and anti-AcH3 antibody. (**B**) H4 histone acetylation levels were determined with a Western blot using an anti-H4 and anti-AcH4 antibody. Different letters on the bars indicate significant differences between each treatment. H3 = histone H3 antibody; AcH3 = acetylated histone H3 antibody; H4 = histone H4 antibody; AcH4 = acetylated histone H4 antibody.

It was reported that TSA causes an increase in global histone H3 and H4 acetylation in plants [14,17,25,26]. To investigate the effect of TSA on histone acetylation in protoplast conditions, Western blot analysis with H3 and H4 acetylation antibodies and H3 and H4 antibodies was carried out in lettuce protoplasts after TSA treatments (Figure 5). After 6 and 12 h of TSA treatments, histone H3 and H4 acetylation in lettuce protoplasts was significantly increased compared to that in the control, and this increase was concentration-dependent. These results indicate that the level of histone H3 and H4 acetylation was increased by TSA from lettuce protoplasts.

### 2.5. Effects of TSA on Expression of Cell Division Regulatory Genes and Formation of Callus

**Figure 6:**
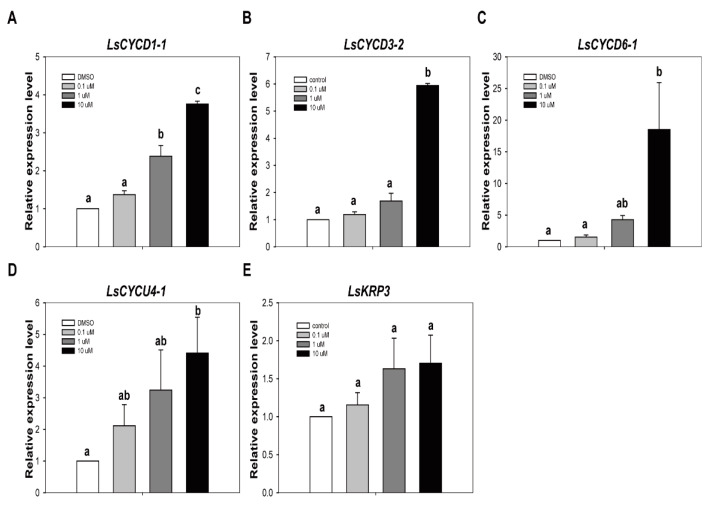
The effect of TSA on expression of cell division regulatory genes. The relative gene expression level of *LsCYCD1-1* (**A**), *LsCYCD3-2* (**B**), *LsCYCD6-1* (**C**), *LsCYCU4-1* (**D**) and *LsKRP3* (**E**) in lettuce protoplasts. qRT-PCR was performed with total RNA extracted from lettuce protoplasts after treatment with DMSO or TSA for 12 hr. Bars represent means ± SE (*n* = 3) of independent experiments. Different letters on the bars indicate significant differences between each treatment (ANOVA with Duncan’s test, *p* < 0.05).

We have already demonstrated that TSA has a stimulatory role in increasing the efficiency of genome editing from protoplasts. Initial cell division and callus formation from protoplast are essential steps for the development of genome-edited plants using the CRISPR/Cas9 RNP system. In order for TSA to be used as a positive stimulator for the development of genome-edited plants, its effect on subsequent cell division and callus formation from protoplasts must be investigated. If TSA prevents subsequent cell division and callus formation from protoplasts, it cannot be used as a positive stimulator for the development of genome-edited plants. Thus, we first checked how TSA treatment affects cell division-related gene expression from lettuce protoplasts. The quantitative reverse transcription PCR (qRT-PCR) analysis showed that the transcripts levels of *LsCYCD1-1*, *LsCYCD3-2*, *LsCYCD6-1*, and *LsCYCU4-1* were increased by TSA treatment, except for *LsKRP3* (Figure 6). Overall, when a high concentration of TSA (10 µM) was treated, cell division-related gene expression was also significantly increased. Compared to the high-concentration TSA treatment, the gene expression was decreased in the low-concentration (0.1 μM) TSA treatment. These results show that the TSA-induced increase in gene expression occurs in a concentration-dependent manner. On the other hand, there was no statistically significant difference in the expression of the cell division inhibitor *KRP3* gene by TSA treatment. Considering these results, we suggest that TSA treatment has a positive effect on the expression of cell division-related genes as well as genome editing efficiency from lettuce protoplasts.

Next, we observed the effects of TSA on callus proliferation from Cas9-transfected tobacco protoplasts (Figure S2). Tobacco protoplasts are actively divided and easily form calluses, making them suitable for checking the effects of TSA treatment during plant regeneration. The green calluses were derived from tobacco protoplasts grown for approximately 5 weeks in a callus induction medium. As shown in Figure S2, callus proliferation from non-transfected tobacco protoplasts was significantly enhanced upon TSA treatment; the size of callus clumps was increased strikingly by treatment with 1 µM TSA. Interestingly, treatment with TSA at 1 μM concentrations also promoted callus proliferation from transfected tobacco protoplasts compared to the control, although not as much as in the case of untreated protoplasts. In addition, *PDS* gene-edited tobacco plants with an albino phenotype were obtained from the green callus. (Figure S3). The result suggests that TSA treatment promotes callus proliferation from the transfected or non-transfected tobacco protoplasts as well as genome editing efficiency.

## 3. Discussion

To date, many studies related to the development of plants with genomes edited using the Cas9 delivery system have been reported [10,27,28,29]. However, most studies are focused on the development of genome-edited plants using the Cas9 vector system. Plant regeneration from protoplasts can be used as an effective alternative for the development of genome-edited plants. However, the low efficiency of RNP delivery into protoplasts and the difficulty of plant regeneration from protoplasts are major limitations for the development of genome-edited plants through direct RNP delivery into protoplasts. Here, we examined the effects of TSA on the genome editing efficiency when PEG transfection was performed using protoplasts with the Cas9 protein-gRNA delivery system. TSA treatment significantly increased the efficiency of indel formation more than 3.5 times in the lettuce *SOC1* gene and 1.8 times in the tobacco *PDS* gene. Although there is a very large variance of indel formation rate between each replicate, the fold difference of indel formation rate was consistent when treated with TSA versus DMSO each alone in three independent experiments. There are several causes, but the major cause of the deviation is probably the status of protoplasts. There is a difference in the amount or viability of freshly isolated protoplasts despite applying the same sample, the same cell wall degrading enzyme, the same incubation temperature and incubation time. In the process of direct RNP transfer into protoplasts, many physical and chemical impacts occur, which are thought to have a significant effect on the stability of protoplasts. In this study, the indel frequencies of the *LsSOC1* and *NbPDS* gene from TSA treatments were about 3.3 to 3.8 times and 1.5 to 1.8 times higher than DMSO treatment, respectively. These improvements can be applied as a means of increasing genome editing efficiency using plant protoplasts. The development of genome-edited plants using direct RNP delivery into protoplasts has the advantage of reducing the effort required to remove the vector sequence in next-generation seeds. DNA cleavage by Cas9 was repaired by non-homologous end joining (NHEJ) predominantly and genetic disruption and gene knockout occurs. It can be used in editing genes that negatively affect the expression of desired traits. On the other hand, homology-directed repair (HDR) can deal with DSB in an error-free manner in the presence of a DNA template with homology to the sequences flanking the DSB. This will allow us to insert the desired DNA sequence and manifest the desired trait. Therefore, our results shown in this study could be used as a means of developing protoplast-based genome-edited plants whether it is DNA repair by NHEJ or by HDR.

Previous studies have shown that TSA increases the efficiency of genome editing in mammalian cells [15]. Until now, there have been no reports of how TSA works in plant protoplasts. Similar to mammalian cells, we presented that the TSA has a stimulatory role in increasing the genome editing efficiency from lettuce and tobacco protoplasts in this study. Considering these results, we inferred that TSA might have a positive effect on genome editing in overall eukaryotic cells including plant cells. However, the enhancement of genome editing by TSA may vary depending on the endogenous acetylation level of the target gene. Therefore, the effects in a particular plant cell on different targets and in a variety of plant cells remain to be further elucidated.

The acetylation levels of histone H3 and H4 were increased in lettuce protoplasts by TSA treatment. An increase in H3 and H4 acetylation seems to have an effect of loosening the chromatin structure by affecting the overall chromatin structure. Although acetylation at a specific residue determines chromatin condensation and de-condensation, overall histone acetylation is known to result in chromatin de-condensation. Therefore, there are many reports of using TSA to bring about chromatin de-condensation [15,30,31,32]. We showed that treatment with TSA increased histone H3- and H4 acetylation and loosened chromatin structures. This can be promoted in the following ways: de-condensation of the overall chromatin might facilitate CAS9 and gRNA to find the target. In a previous study, it was shown that closed chromatin and methylated DNA negatively impact Cas9 binding, while an increased abundance of Cas9 protein-gRNA complexes and guide sequences in the genome positively impact Cas9 binding [33]. A strong correlation between Cas9-bound sites and open chromatin was already reported in [34]. The changes in global chromatin including the target DNA sequences can bring out increased accessibility, leading to increased genome editing. In other words, TSA treatment allows chromatin and Cas9 protein-gRNA RNPs to gain more access to their targets, bringing out more cuts detected in open chromatin. Another possibility is that DNA damage repair machinery can easily access DNA double strands cut by Cas9. Because eukaryotic DNA is packaged into nucleosomes, the structural units of chromatin, chromatin modification is necessary during DNA damage repair and is achieved by histone modification and chromatin remodeling. Cas9 protein cleaves the target region in a sequence-dependent manner, and DSB is generated and repaired by endogenous DSB repair machinery in that region, resulting in genome modification. The proper recruitment of DSB repair machinery to DSB could help promote genetic modification. Moreover, it was already known that TSA activates ATM-dependent DNA repair pathways. Ultimately, genome editing can be promoted by increased homologous recombination [35].

Although the chromatin accessibility of TSA treatment groups was higher than that of the DMSO treatment group, the changes in chromatin structure with the TSA treatment concentration appear weak from lettuce protoplasts. The reason for the low concentration dependence may be because the entire chromatin shows subtle changes. In previous reports, when other HDAC inhibitors were treated with higher concentrations, chromatin changes were clearly increased [36]. However, treatment with high concentrations of TSA may have an inhibitory effect on cell division [37,38,39,40,41]. Therefore, determination of an appropriate concentration of TSA is necessary without inhibition of cell division for the development of genome-edited plant from the protoplast. In addition, since the optimal concentration for TSA treatment may differ for each crop or for each target, careful consideration is needed to determine the appropriate concentration for TSA treatment.

We examined the effects of TSA on expression of cell division regulatory genes and callus formation. The results suggest that TSA increases cell division-related gene expression, ultimately helping to progress smoothly from the protoplast to the callus stage. Many HDAC inhibitors including TSA have been applied in the plant regeneration process to increase regeneration efficiency. The totipotency in the male gametophyte of *Brassica napus* is promoted by TSA treatments [17] and the rate of microspore embryogenesis and the frequency of direct plant regeneration are increased by treatments of HDAC inhibitors, TSA and SAHA, in pakchoi [18]. Similarly, TSA promotes microspore-derived embryogenesis and regeneration of green wheat plants, suggesting that TSA leads to an increase in histone acetylation and in global alteration of gene expression [19]. Similar to TSA, NaB, an HDAC inhibitor, was reported to increase the embryo induction rate and percentage of embryos that directly regenerate to plants in *Brassica rapa* [18]. It was also reported that NaB treatment facilitates adventitious shoot formation from tobacco protoplast-derived calluses [10]. Taken together, TSA treatment has a positive effect on expression of cell division-related genes, leading to efficient plant regeneration as well as genome editing efficiency from protoplasts. However, the optimal concentration and treatment time of TSA for generation of genome-edited plants can be different depending on plant species.

Although TSA promotes cell division and callus induction, it might adversely affect plant development and reproduction resulting from epigenetic traits. This is because TSA is a potent HDAC inhibitor that can induce serious damage to cells or plants. High concentration of TSA treatment may have an inhibitory effect in initial cell division from lettuce and tobacco protoplasts rather than stimulatory effect. Furthermore, shoots derived from TSA-treated protoplasts could not be converted into normal plantlets or would show any morphological abnormality. The highest concentration of TSA treatment is expected to create serious damage to the generated plantlets. Therefore, it is necessary to continue research on how it affects plant development after callus, and further research is needed on the effect of treatment with relatively weak HDAC inhibitors such as SAHA or valproic acid (VPA) [42]. Since it was observed that the increase in the genome editing efficiency during plant gene editing is increased even with the low concentration TSA treatment, it seems desirable to use the low concentration TSA treatment to increase the genome editing rate and reduce toxic side effects. If we easily obtain genetically modified plants by increasing the genome editing efficiency by low-concentration TSA treatment, this will be of great help in addressing plant-related global issues, such as strengthening the global food supply, responding to global warming, and sustainable agriculture.

It has been reported that treatment with the histone deacetylase inhibitor VPA increases the genome editing efficiency in mouse embryonic stem cells and embryos [42]. In addition, the positive effect of NaB as well as VPA on CRISPR/Cas9 cutting efficiency has been reported in hematopoietic stem and progenitor cells [43]. Other HDAC inhibitors as well as TSA appear to play a positive role in the genome editing process in plant cells. For example, when maize cells were genome-edited using a non-functional GPF vector reporting system, not an RNP system using Cas9, treatments with NaB or nicotinamide increase GFP positive cells, suggesting these two inhibitors promote genome editing [36]. It seems that other HDAC inhibitors could be also used using the RNP system with plant protoplasts because those also increase the accessibility of gRNA by releasing chromatin or increase the accessibility of DNA damage repair machinery to Cas9 induced DNA cleavage sites. It seems necessary to study and compare the effect of other HDAC inhibitors on genome editing efficiency and plant regeneration process forms protoplasts and select the optimal HDAC inhibitor suitable for each plant type.

Epigenetic regulation is very important for plant development and regeneration. Epigenetic reprogramming during de novo organogenesis of plant tissue culture is relatively well known [44,45,46]. For example, TSA promoted the formation of callus and embryo-like structures from leaf explants of *Arabidopsis* [47,48]. We have already reported that the effect of sodium butyrate, one of HDAC inhibitors, on in vitro adventitious shoot formation is different for each plant species or for each type of plant tissue [49]. In this study, we reported that TSA treatment is able to increase the genome editing efficiency using lettuce and tobacco protoplasts. This is the first successful report showing that TSA has a stimulatory role in increasing editing efficiency through chromatin structural modification from plant cells in a similar manner to mammal cells. Although TSA treatment increases the genome editing efficiency, if TSA prevents cell division and callus proliferation from protoplasts, it could not be used to obtain whole plants. In the development of genome-edited plants using the CRISPR/Cas9 RNP system, initial cell division and callus formation from protoplasts are critical bottlenecks to be overcome for successful plant regeneration. Fortunately, we also confirmed that treatment of TSA can effectively improve the expression of cell division regulatory genes and callus proliferation from PEG-transfected protoplasts. In conclusion, our results could contribute to increasing the efficiency of CRISPR/Cas9-mediated genome editing with plant cells. Furthermore, it could be applied for the development of useful genome-edited crops using the CRISPR/Cas9 system with plant protoplasts.

## 4. Materials and Methods

### 4.1. Plant Materials, Protoplast Isolation and PEG Transfection

The tobacco (*Nicotiana benthamiana* L.) and lettuce (*Lactuca sativa* L.) cv Cheongchima [9] seeds were sterilized with 70% ethanol for 3 min and sterilized with 1% Sodium hypochlorite (commercial *Clorox*) for 10 min. The sterilized seeds were washed five times using sterile water and placed on MS medium (1/2 MS basal salts, 0.4 mg L^−1^ thiamine, 100 mg L^−1^ myo-inositol, 30 g L^−1^ sucrose, 4 g L^−1^ gelrite, pH 5.7) for seed germination. Tobacco and lettuce seeds were grown for 4 weeks; 1 week at 25 °C, 20 °C, under light culture conditions (light period: 16/8 h, light intensity: 80 μmol m^−2^ s^−1^) in a growth chamber. The green stem cut from the germinated tobacco plant was transferred to a medium (MS basal salts, 100 mg L^−1^ myo-inositol, 0.4 mg L^−1^ thiamin, 0.5 mg L^−1^ kinetin, 0.1 mg L^−1^ IBA, 30 g L^−1^ sucrose, 0.8 mg L^−1^ plant agar, pH 5.7) to induce mass growth of the plant in vitro. Tobacco stems grown in vitro were transferred to a solid medium of the same composition at about 4 weeks intervals and cultured. Later, the isolation of tobacco protoplasts was conducted using leaves of tobacco plants grown in vitro.

Protoplasts were isolated from the lettuce seedlings as previously described with some modifications [10]. For protoplast isolation, the leaves of tobacco plants and cotyledons of 7 d lettuce seedlings were digested with enzyme solution (1% Viscozyme (Viscozyme L, Novozyme), 0.5% Celluclast (C2730, Novozyme), 0.5% Pectinex (33095, Novozyme), 9% mannitol, 3 mM MES, CPW solution [50], pH 5.7) during incubation with shaking (40~50 rpm) for 4~6 h at 25 °C in darkness. The mixture was filtered before protoplasts were collected by centrifugation at 114× *g* in a round-bottomed tube for 5 min. The purified protoplasts were washed with W5 solution (2 mM MES, 154 mM NaCl, 125 mM CaCl_2_, 5 mM KCl, pH 5.7) and pelleted by centrifugation at 114× *g* for 5 min. Finally, protoplasts were resuspended in W5 solution and counted under the microscope using a hemocytometer. Protoplasts were diluted to a density of 1 × 10^6^ protoplasts/mL of MMG solution (0.4 M mannitol, 15 mM MgCl_2_, 4 mM MES, pH 5.7).

Cas9 protein (3 μg) and in vitro synthesized gRNA (1 μg) were mixed in 1× NEB buffer 3 for at least 10 min in advance to form RNP complexes. A total of 1 × 10^6^ protoplast cells in a 200 μL MMG solution were mixed gently with the RNP complexes and 200 μL PEG solution (40% *w*/*v* PEG 4000, 0.2 M mannitol, 0.1 M CaCl_2_) and incubated for 10 min. Then, 400 μL W5 solution was added and mixed carefully and incubated for 10 min. Additional 800 μL W5 solution was added and protoplasts were collected by centrifugation at 55× *g* for 5 min. The tobacco protoplasts were resuspended gently in 1 mL of protoplast culture medium (B5 [51] (Gamborg including vitamins) salts, 60 g L^−1^ myo-inositol, 2 mg L^−1^ BA, 0.5 mg L^−1^ NAA, 20 g L^−1^ sucrose, pH 5.7), and lettuce protoplasts were resuspended gently in 1 mL of protoplast culture medium (MS salts, 0.4 mg L^−1^ thiamine, 100 mg L^−1^ myo-inositol, 30 g L^−1^ sucrose, 0.2 mg L^−1^ 2,4-D, 0.3 mg L^−1^ BA, pH 5.7) and cultured under dark conditions at 25 °C for 48 h to analyze genome editing efficiency.

### 4.2. TSA Treatment and Protoplast Culture

After the protoplast PEG transfection, they were suspended in the protoplast culture medium in a 60 × 15 mm Petri dish (Falcon, 3002). TSA was added to the 2 mL protoplast culture medium per dish immediately after the PEG transfection of protoplasts and the culture dishes were incubated at 25 °C in the dark. Cell division from protoplasts was periodically examined under a microscope during the protoplast culture. TSA (Sigma, T8552) was dissolved in dimethyl sulfoxide (DMSO) for stock preparation. The stock solutions were sterilized by filtration and were stored at −20 °C until use. We treated protoplasts with the concentration of 0, 0.1, 1 and 10 μM TSA to show an enhanced effect. Each treatment was performed immediately after PEG transfection.

### 4.3. gRNA Design and Synthesis

The Cas9 protein was purchased from ToolGen, Inc. (Seoul, Korea). Guide RNAs were transcribed in vitro by Precision gRNA Synthesis Kit (Invitrogen, A29377) according to the manufacturer’s protocol. In vitro cleavage assay was carried out to test if transcribed sgRNAs work well according to the ToolGen manufacturer’s protocol. Primer lists for guide RNA transcription are presented in Supplementary Table S2.

### 4.4. Deep Seq

Genomic DNA from protoplasts 48 h after PEG transfection was prepared by AccuPrep^®^ Genomic DNA Extraction Kit (BIONEER, K-3032) according to the manual. The target sites were amplified from the genomic DNA with PCR primer and sequencing adaptors (Supplementary Table S2). High-throughput sequencing was performed using Illumina MiSeq (v2, 300-cycle).

### 4.5. MNase Assay

MNase assays were performed as previously described [39]. MNase-digested chromatin DNA was electrophoresed on 1.5% agarose gels and visualized by staining with EcoDye™ Nucleic Acid Staining Solution (Biofact, ES301-1000). The genomic band intensities without (g_0_) and with (g_c_) treatment with different concentrations of MNase were quantified using Image J software (NIH, Bethesda, MD, USA). The ratio of g_c_/g_0_ was used to represent the degree of chromatin relaxation.

### 4.6. Histone Protein Extraction and Western Blot

Histone extraction was performed using Abcam’s protocol (https://www.abcam.com/protocols/histone-extraction-protocol-for-western-blot) with slight modifications. The protoplasts cultured in the protoplast culture medium (PCM) containing 0, 0.1, 1 and 10 μM of TSA. After 6 or 12 h of incubation, protoplasts were harvested and re-suspended in tritone extraction buffer (TEB; PBS containing 0.5% Triton × 100 (*v*/*v*), 2 mM phenylmethylsulfonyl fluoride (PMSF)). Cells were lysed on ice for 10 min and centrifuged at 6500× *g* for 10 min at 4 °C to spin down the nuclei. Then, we discarded the supernatant and washed the pellet in TEB and centrifuged as before. The histones were extracted from re-suspended the pellet in 0.2 N HCl overnight at 4 °C. The supernatants were collected and neutralized with 0.1 volume of 2 N NaOH. Histones were separated by 15% sodium dodecyl sulfate polyacrylamide gel electrophoresis (SDS-PAGE) and detected using a specific antibody against histone H3 (Abcam, ab1791), acetylated histone H3 (Merck Millipore 06–599, Burlington, Massachusetts, USA), histone H4 (Merck Millipore 05-858), and acetylated histone H4 (Merck Millipore 06-866) by Chemiluminescence system Fusion Solo S (Vilver, France). The ratio of detected histones was calculated using built-in software.

### 4.7. Formation of Callus Proliferation from Protoplast-Derived Cells

TSA was added to the 2 mL protoplast culture medium per dish immediately after the PEG transfection of protoplasts and the tobacco protoplasts treated with TSA or DMSO were cultured at 25 °C in the dark for 4 weeks. Then, 10 mL of liquid protoplast culture medium was added and transferred to a 16 h light/8 h dark photoperiod (30 μmol m^−2^ s^−1^) and further cultured at 25 °C with shaking at 50 rpm. After 4 weeks of culture, the micro-calluses were transferred to the regeneration medium (MS salts, 30 g L^−1^ sucrose 6 g L^−1^ gelrite, 0.1 mg L^−1^ NAA, 0.5 mg L^−1^ BA, pH 5.7) for callus proliferation. The callus clumps were observed after about 4~5 weeks of culture on the regeneration medium. For callus proliferation, three independent replicate experiments were carried out.

### 4.8. Statistical Analysis

Data analyses were performed using the SPSS software, and the averages with the standard deviations were compared by one-way ANOVA with Duncan’s test (*p* < 0.05). Different letters in the figures indicate significant differences among the samples at a threshold of *p* < 0.05. Bars in all figures represent means ± SD determined from over three biological replicates. The number of experiments performed is indicated by the number n in the figure.

## 5. Conclusions

Based on our findings using lettuce and tobacco protoplasts, Trichostatin A (TSA), a representative histone deacetylase (HDAC) inhibitor, increases CRISPR/Cas9-mediated genome editing efficiency, perhaps by increasing the gRNA’s access to the target and the DNA repair machinery’s access to DSB due to chromatin de-condensation in plants. Furthermore, treatment with TSA can effectively improve the expression of cell division regulatory genes and callus proliferation using PEG-transfected protoplasts. These findings could contribute to increasing the efficiency of genome editing and applied for the development of useful genome-edited crops using the CRISPR/Cas9 system with plant protoplasts. Furthermore, this technique can be refined for optimum outcomes in editing efficiency and regeneration for various plant species in the future.

## Figures and Tables

**Table 1:** Summary of the indel frequencies based on deep sequencing analysis of *LsSOC1* target region from lettuce protoplasts. Each treatment consisted of three repeats, and average and standard deviation are represented. The superscripts in indel frequency indicate significant differences between each treatment (ANOVA with the Duncan’s test, *p* < 0.05).

Plant Species	Concentration of TSA (μM)	Total Reads	WT	Insertions	Deletions	Indel Frequency (%)
*L. sativa*	0 (DMSO)	54,379 ± 3823	53,427 ± 4239	788 ± 283	165 ± 128	1.8 ± 0.9 ^a^
	0.1	50,396 ± 1629	47,864 ± 16,395	2179 ± 93	353 ± 126	5.6 ± 2.6 ^b^
	1	55,397 ± 20,223	52,292 ± 16,785	2677 ± 571	428 ± 176	6.2 ± 3.2 ^b^
	10	53,667 ± 3833	50,610 ± 20,077	2639 ± 322	419 ± 101	6.4 ± 2.8 ^b^

## Data Availability

All datasets for this study are included in the manuscript and the supplementary files.

## Supplementary Materials

The following are available online at https://www.mdpi.com/article/10.3390/ijms22157817/s1, Figure S1: The changes in gene editing efficiency according to TSA treatment in tobacco, Figure S2: The effect of TSA on callus proliferation from the transfected or non-transfected tobacco protoplasts, Figure S3. Representative images of PDS gene-edited tobacco plants from the transfected green calluses. A full list of the detected mutations by targeted deep sequencing is provided in the Supplementary excel data.
