# Supplementary material for: TSA Promotes CRISPR/Cas9 Editing Efficiency and Expression of Cell Division-Related Genes from Plant Protoplasts
Source: Int J Mol Sci. 2021 Jul 22;22(15):7817. doi: 10.3390/ijms22157817 (PMC8346083; doi:10.3390/ijms22157817)

*Article*

# TSA Promotes CRISPR/Cas9 Editing Efficiency and Expression of Cell Division-Related Genes from Plant Protoplasts

**Seung Hee Choi[1], Myoung Hui Lee[1], Da Mon Jin[1,2], Su Ji Ju[1,3], Woo Seok Ahn[1,2], Eun Yee Jie[1], Ji Min Lee[1], Jiyoung Lee[1], Cha Young Kim[1] and Suk Weon Kim[1,*]**

[1] Biological Resource Center, Korea Research Institute of Bioscience and Biotechnology, 181 Ipsingil, Jeongeup-si, Jeollabuk-do, 580-185, Korea; csh@kribb.re.kr (S.H.C.); mhlee17@kribb.re.kr (M.H.L.); damon@kribb.re.kr (D.M.J.); dntjr0412@kribb.re.kr (W.S.A.); jeannie@kribb.re.kr (E.Y.J.); jimin1215@kribb.re.kr (J.M.L); jiyoung1@kribb.re.kr (J.L); kimcy@kribb.re.kr (C.Y.K)

[2] Department of Bioenergy Science and Technology, Chonnam National University, Gwangju 500-757, Korea

[3] Department of Applied Plant Science, Chonnam National University, Gwangju 500-757, Korea; suji@kribb.re.kr (S.J.J.)

* Correspondence: kimsw@kribb.re.kr; Tel.: +82-63-570-5650; Fax: +82-63-570-5609

**Figure S1.** The changes in gene editing efficiency according to TSA treatment in tobacco. (**A**) The schematic illustration of *NbPDS* CRISPR/Cas9 target site. The target region is shown in green letters followed by PAM (NGG; red). (**B**) Relative efficiency of indel frequency (%) of at target site in protoplasts examined at 48hr after transfection of Cas9 and gRNAs as RNP complexes with DMSO or TSA treatment. The indel frequency of the DMSO treatment group was set to 100%, and those of the TSA treatment groups were shown relatively. Bars represent means ± SE (n = 3) of independent experiments. Different letters on the bars indicate significant differences between each treatment (ANOVA with the Duncan's test, $p < 0.05$).

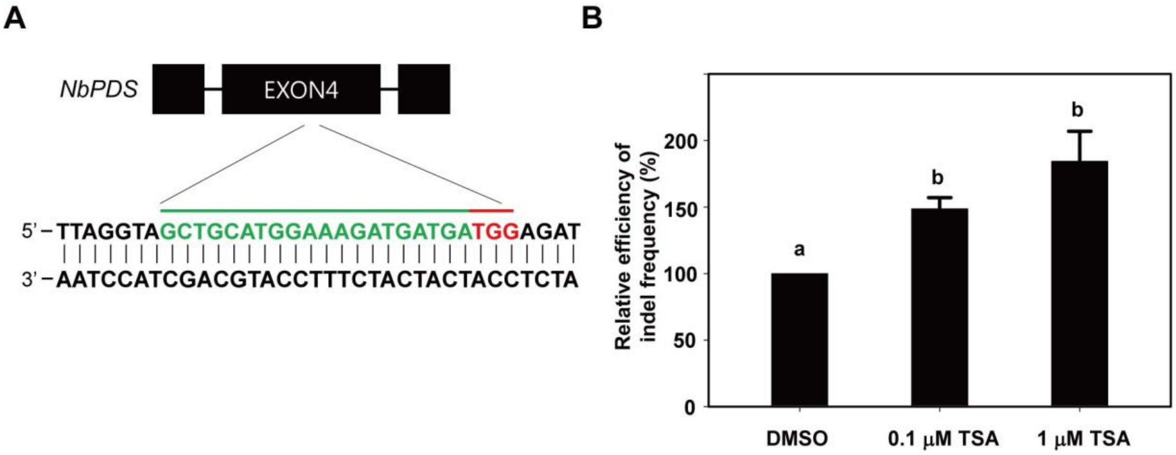

**Table S1.** Mutation rates based on deep sequencing of *NbPDS* target region.

| Plant species | # | Concentration of TSA (uM) | Total reads | WT | Insertions | Deletions | In-del frequency (%) |
|---|---|---|---|---|---|---|---|
| *N. benthamiana* | 1ST | 0 (DMSO) | 63,162 | 62,562 | 259 | 341 | 600 (0.9%) |
| | | 0.1 | 49,324 | 48,678 | 247 | 399 | 646 (1.3%) |
| | | 1 | 43,538 | 42,676 | 459 | 403 | 862 (2.0%) |
| | 2nd | 0 (DMSO) | 61,563 | 59,689 | 515 | 1,359 | 1,874 (3%) |
| | | 0.1 | 37,193 | 35,667 | 565 | 961 | 1,526 (4.1%) |
| | | 1 | 50,570 | 47,754 | 792 | 2,024 | 2,816 (5.6%) |
| | 3rd | 0 (DMSO) | 23,498 | 22,239 | 573 | 686 | 1,259 (5.4%) |
| | | 0.1 | 23,710 | 21,604 | 879 | 1,227 | 2,106 (8.9%) |
| | | 1 | 38,027 | 35,067 | 1,454 | 1,506 | 2,960 (7.8%) |

**Table S2.** The primers that were used in this study

| Primer name | Target | Sequence (5'→3') | No. bases |
|---|---|---|---|
| *LsSOC1* 1ST PCR F | *LsSOC1* | TAGTCCACACACTCCATCGC | 20 |
| *LsSOC1* 1ST PCR R | *LsSOC1* | AGGAAAAGAACCCACAAACCAG | 22 |
| *LsSOC1* adapter PCR F | *LsSOC1* | AAGAAGACTTGACATTTGATTGGT | 24 |
| *LsSOC1* adapter PCR R | *LsSOC1* | TGGAGAAGGTCACTTGTCTACTTG | 24 |
| *LsSOC1* gRNA F | *LsSOC1* | taatacgactcactatagGAGGGAAGACTCAAAT | 34 |
| *LsSOC1* gRNA R | *LsSOC1* | ttctagctctaaaacCCGCATTTGAGTCTTCCCT | 34 |
| *LsSOC1* genomic PCR F | *LsSOC1* | TGTAACTTGAACATGCGACCATAC | 24 |
| *LsSOC1* genomic PCR R | *LsSOC1* | CATTATAAGGAAAAGAACCCACAAAC | 26 |
| *LsCyclinD1-1* qF | *LsCyclinD1-1* | TCTCGAACCAACGAACTCCA | 20 |
| *LsCyclinD1-1* qR | *LsCyclinD1-1* | CTCTCCGGTACATCCACTCC | 20 |
| *LsCyclinD3-2* qF | *LsCyclinD3-2* | ACCCATTACGGATTCGTTGC | 20 |
| *LsCyclinD3-2* qR | *LsCyclinD3-2* | TTGCAGGTCGAGGAGAAGAG | 20 |
| *LsCyclinD6-1* qF | *LsCyclinD6-1* | CGGGAAGCGTTGACAGTTAT | 20 |
| *LsCyclinD6-1* qR | *LsCyclinD6-1* | GCGAGAGAAAGGCACGAAAT | 20 |
| *LsCyclinU4-1* qF | *LsCyclinU4-1* | TCAAGTACGCCAATTGTAGCC | 21 |
| *LsCyclinU4-1* qR | *LsCyclinU4-1* | CATCCATGAACTTGGCAGCA | 20 |
| *LsKPR3* qF | *LsKPR3* | CCGTCATGACCAGGAACAAC | 20 |
| *LsKPR3* qR | *LsKPR3* | AGCGGAATTACACCAATGCC | 20 |
| *NbPDS* 1ST PCR F | *NbPDS* | CCTGTTGGTTGCATTTCTCA | 20 |
| *NbPDS* 1ST PCR R | *NbPDS* | GAAAATCAAAGCGGCTGAAC | 20 |
| *NbPDS* adapter PCR F | *NbPDS* | CTGAAGCAGTCACCAAGAATC | 21 |
| *NbPDS* adapter PCR R | *NbPDS* | GTGCAACCCAGTCTCGTACC | 20 |
| *NbPDS* gRNA F | *NbPDS* | taatacgactcactatagGCTGCATGGAAAGATG | 34 |
| *NbPDS* gRNA R | *NbPDS* | ttctagctctaaaacTCATCATCTTTCCATGCAG | 34 |
| *NbPDS* genomic PCR F | *NbPDS* | GAGTCAATTTTACCCGTCCTGTTG | 24 |
| *NbPDS* genomic PCR R | *NbPDS* | GAATGTTCCTTCCACTGCAACC | 22 |

**Figure S2.** The effect of TSA on callus proliferation from the transfected or non-transfected tobacco protoplasts. Representative images of callus development after DMSO or TSA treatment during tobacco protoplast cultures. Scale bars: 1 cm.

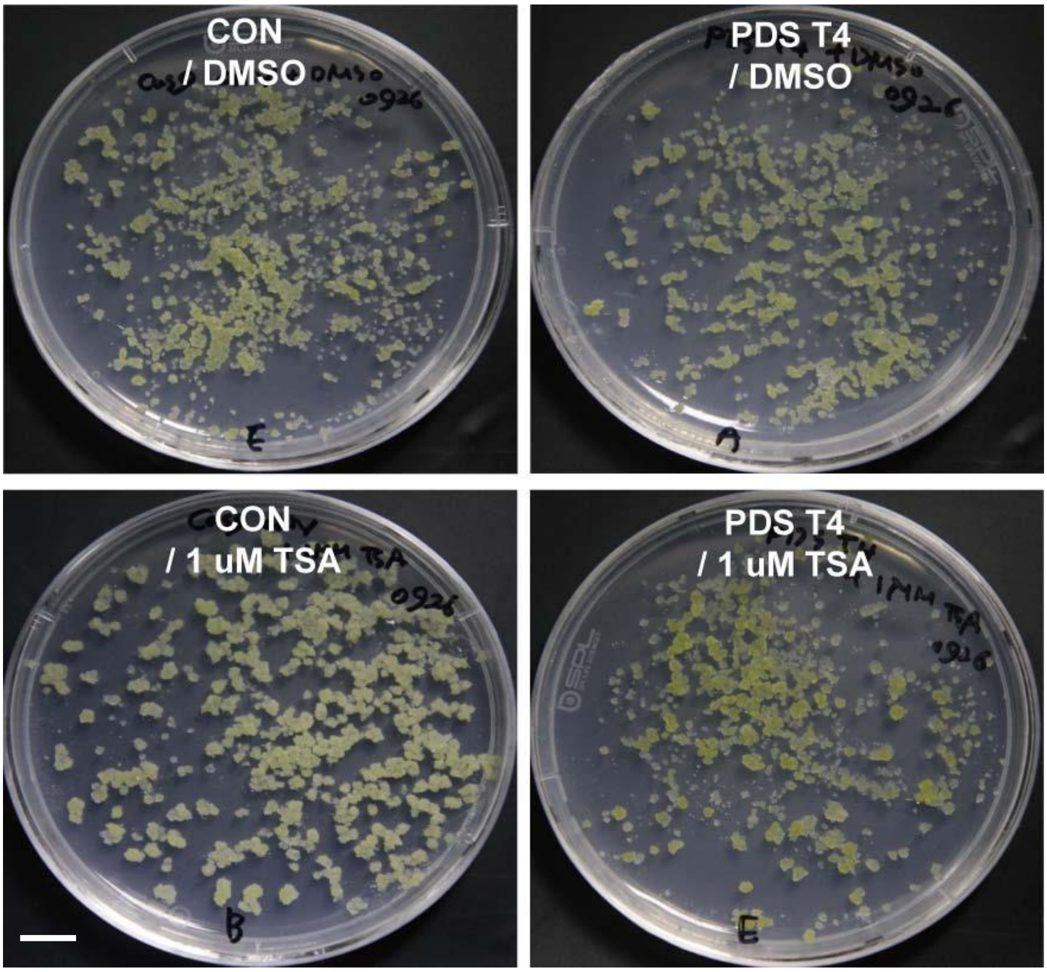

**Figure S3.** Representative images of *PDS* gene-edited tobacco plants from the transfected green calluses. Scale bars: 1 cm.

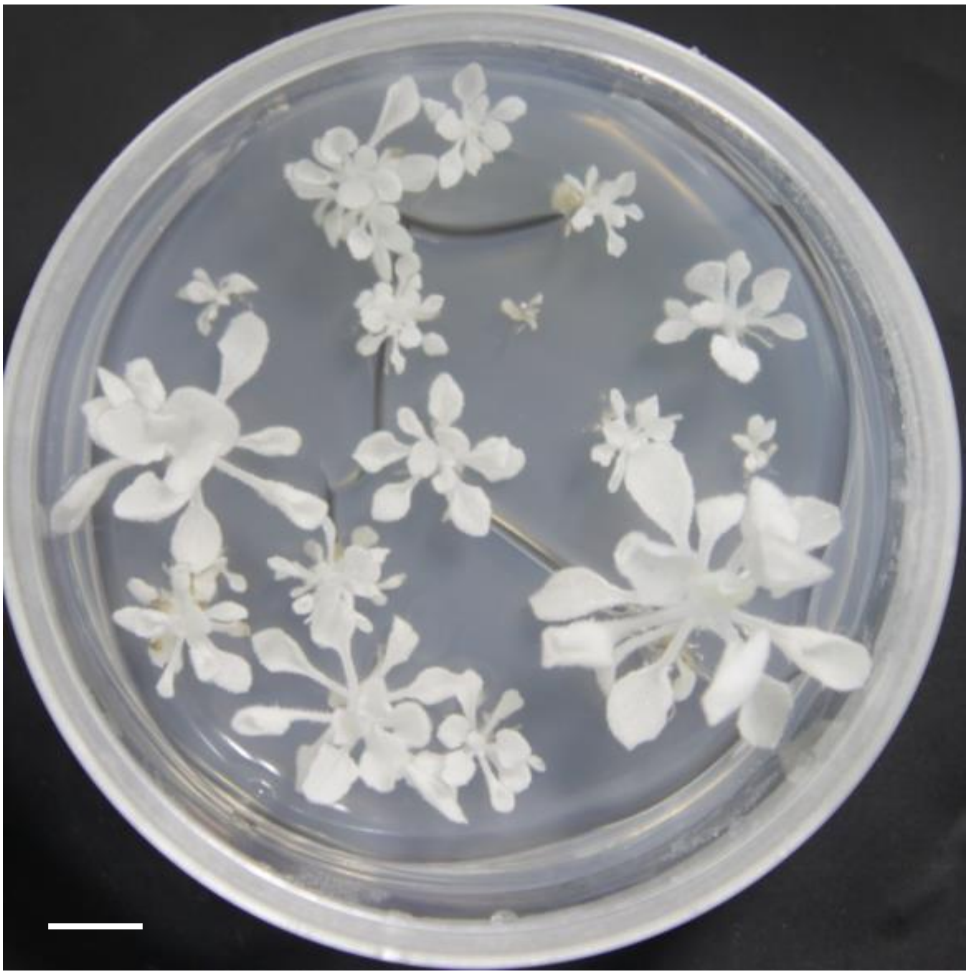

Supplement: Supplementary file 1 [file ijms-22-07817-s001.zip › Supplementary-R.pdf]
